# Impaired ribosome biogenesis: mechanisms and relevance to cancer and aging

**DOI:** 10.18632/aging.101922

**Published:** 2019-04-26

**Authors:** Zsofia Turi, Matthew Lacey, Martin Mistrik, Pavel Moudry

**Affiliations:** 1 Institute of Molecular and Translational Medicine, Faculty of Medicine and Dentistry, Palacky University, 779 00 Olomouc, Czech Republic

**Keywords:** ribosome biogenesis, ribosomopathy, aging, cancer, p53

## Abstract

The biosynthesis of ribosomes is a complex process that requires the coordinated action of many factors and a huge energy investment from the cell. Ribosomes are essential for protein production, and thus for cellular survival, growth and proliferation. Ribosome biogenesis is initiated in the nucleolus and includes: the synthesis and processing of ribosomal RNAs, assembly of ribosomal proteins, transport to the cytoplasm and association of ribosomal subunits. The disruption of ribosome biogenesis at various steps, with either increased or decreased expression of different ribosomal components, can promote cell cycle arrest, senescence or apoptosis. Additionally, interference with ribosomal biogenesis is often associated with cancer, aging and age-related degenerative diseases. Here, we review current knowledge on impaired ribosome biogenesis, discuss the main factors involved in stress responses under such circumstances and focus on examples with clinical relevance.

## Introduction

The nucleolus has gained prominent attention in molecular research over the past two decades, due to its emerging role in various cellular processes. Among them, the production of ribosomes is seemingly the most important, as it controls translation of all proteins in the cell and thus governs cell growth and proliferation [1]. The nucleolus is a subnuclear, membrane-less organelle, formed in early G1 phase of the cell cycle around the short arms of acrocentric chromosomes (chromosome 13, 14, 15, 21 and 22), in nucleolar organizer regions (NORs). These NORs contain the ribosomal DNA (rDNA) genes, arranged in tandem repeats and transcribed by RNA Polymerase I (Pol I) [2]. The resulting single polycistronic transcript, known as 47S pre-rRNA, is further modified in the nucleolus. The maturation of the primary transcript is initiated co-transcriptionally and the main processing steps involve endo- and exonucleolytic cleavages, pseudouridylation and 2’-O-methylation which lead to the emergence of three ribosomal RNA (rRNA) species: 18S, 5.8S and 28S rRNAs. While 18S rRNA is in-corporated into the small ribosomal subunit (SSU), 5.8S and 28S rRNAs, along with 5S rRNA, are members of the large ribosomal subunit (LSU) [3]. The gene encoding 5S rRNA is an exception when compared to other rRNA genes as it is located on chromosome 1 and transcribed by RNA Polymerase III (Pol III) in the nucleus [4, 5]. The protein components of the ribosome are 80 ribosomal proteins (RPs), which are transcribed in the nucleus by RNA Polymerase II (Pol II) and translated in the cytoplasm. However, both the 5S rRNA and the RPs need to be imported into the nucleolus in order to be incorporated into the ribosome [6]. During late ribosome maturation, the forming subunits are first moved into the nucleus, followed by transport to the cytoplasm where ribosomes can fully assemble and assume their protein-translation function [3].

It can be readily accepted that ribosome biosynthesis consumes most of the cell’s energy, particularly when compared to other biological processes, as it requires the synthesis of the most abundant RNA and protein species in the cell. This not only includes the concerted action of all three RNA polymerases and the cell’s translation apparatus, but also the activity of more than 200 non-ribosomal factors within the nucleolus [7, 8]. Therefore, it is not surprising that cellular signaling networks which sense the nutrient status, growth factors, extra- and intracellular stress levels affect the rate of ribosome biogenesis, mainly by altering the activity of Pol I [9, 10]. Disruption of ribosome biogenesis also promotes signaling pathways that lead to cell cycle arrest and cellular senescence or apoptosis [8, 11]. The earliest observation that impaired ribosome biogenesis halts cell cycle progression comes from a study by Volarevic et al., where they described that the conditional knockout of ribosomal protein RPS6 (eS6) causes cell cycle arrest in mouse liver cells [12]. Since then, a number of studies have demonstrated that the disruption of virtually any step in ribosome biogenesis can result in cell cycle arrest, primarily through activation of the tumor suppressor protein p53. This particular process was recently termed as the Impaired Ribosome Biogenesis Checkpoint (IRBC) [13].

Impaired ribosome biogenesis is usually best visible as structural alterations of the nucleolus which can be seen also in various human diseases [14-17]. Importantly, increased size of nucleoli usually reflects intense ribosome biogenesis and has been recognized by physicians for a long time as a hallmark of many tumor types [18]. Interestingly, despite excessive ribosome biogenesis being believed to drive the fast proliferation of cancer cells, some of the most rapidly dividing tumor cells do not display this phenotype [19]. Moreover, patients with another group of human diseases called ribosomopathies, are prone to developing various kinds of tumors. Ribosomopathies are characterized by mutations in RPs or ribosome biogenesis factors, showing a decreased rate of ribosome biosynthesis due to deficiencies of these components required in the ribo-some biogenesis pathway. Symptoms of these disorders arise from tissue specific growth arrest and/or incompetent translation. There is a wide spectrum of phenotypes displayed by ribosomopathy patients and affected tissues frequently show upregulation of p53 as a consequence of IRBC [20, 21]. Altered ribosome bio-genesis was also connected to aging and it is also relevant in neurodegenerative disorders such as: Alzheimer, Parkinson, Huntington and other advanced age-related diseases (for more details on this topic see the following reviews [16, 17]). However, the exact contribution of IRBC to these complex disorders and aging remains an intriguing question open to further research.

In this review, we summarize the most important steps of ribosome biogenesis, focusing mainly on human cell culture studies. Furthermore, we describe the main effectors of IRBC and review studies that provide evidence for the existence of this pathway as well as examining the clinical relevance of IRBC in aging and age-related diseases.

## Ribosome biogenesis

Ribosome biogenesis begins with rRNA synthesis in the nucleolus. As a first step a pre-initiation complex (PIC) is formed around the rDNA promoter region. The PIC itself consists of the upstream binding factor (UBF), selectivity factor (SL1 also known as TIF1-B), transcription initiation factor 1A (TIF1-A or hRRN3) and Pol I. UBF marks the promoter regions by binding as a homodimer to the core promoter surrounding the transcription start site and to the upstream core element (UCE), thereby creating a DNA loop structure. Next, SL1 is recruited to the promoter: binding to both UBF and the rDNA. The interaction of TIF1-A with Pol I is essential for its recruitment to the promoter and formation of the complete PIC. Promoter opening and escape is also stimulated by UBF and is accompanied by the release of TIF1-A from the Pol I complex [22,23]. Surprisingly, UBF was shown to bind the whole length of rDNA transcript units, and it has been suggested that it is involved in the control of elongation process as well [24]. Transcription termination occurs when Pol I encounters transcription termination factor 1 (TTF-1)-bound terminator elements, the stalled Pol I is subsequently removed by the polymerase I and transcript release factor (PTRF) [25].

In contrast to the synthesis of 47S rRNA, the precursor of 5S rRNA is transcribed by Pol III in the nucleoplasm. The main factors involved in this process are the transcription factors IIIA, IIIB and IIIC (TFIIIA, TFIIIB and TFIIIC), which are responsible for labeling of the promoter region and the recruitment of Pol III [5,26].

The rate of ribosome production is regulated mainly on the level of rRNA synthesis. This is carried out by a number of factors and signaling pathways which are dependent on various cellular needs, such as the availability of nutrients, and the presence of mitogenic or stress signaling [10]. Mitogenic stimuli activate several, typically oncogenic pathways which upregulate rDNA transcription. For example, MAPK/ERK pathway phosphorylates UBF, TIF1-A and TFIIIB to stimulate Pol I and Pol III mediated rRNA transcription, respectively [27-30]. Moreover, both MAPK/ERK and PI3K/AKT signaling activate the expression of c-Myc [31,32], which can boost ribosome biogenesis at multiple levels. It stimulates the formation of PIC by recruiting SL1 to the rDNA promoter, increasing the activity of Pol II to drive transcription of RP genes while simultaneously upregulating Pol III transcription by activating TFIIIB [33-35]. Furthermore, growth factors also activate the mammalian target of rapamycin (mTOR) signaling network which contributes to the activation of UBF, TIF1-A and Pol III associated transcription factors TFIIIB and TFIIIC [36-38]. Additionally, p53 is also involved, both directly and indirectly, in the control of Pol I transcription. It interacts with SL1 to prevent its recruitment to rDNA promoters, thus inhibiting Pol I transcription [39], and also limits Pol III activity via the direct binding of TFIIIB [40]. One of the main transcriptional targets of p53 is p21, which is able to activate the retinoblastoma protein (pRb) through the inhibition of CDKs [41,42]. Besides its well-known role in cell cycle regulation, pRb is able to bind to several ribosome biogenesis factors, like UBF and TFIIIB to suppress rRNA transcription [43-45].

Transcription of rDNA results in the emergence of a single polycistronic primary transcript, known as the 47S rRNA. This transcript contains 18S, 5.8S and 28S rRNAs separated by internal transcribed spacers (ITS1 and ITS2) and flanked by external transcribed spacers (5’-ETS and 3’-ETS). Over the course of rRNA maturation, the ITSs and ETSs are removed by the combined action of endo- and exonucleases. The processing of the 47S pre-rRNA is initiated co-transcriptionally by the formation of the so-called small subunit (SSU) processome [3]. The recruitment of the transcriptional U three protein (t-UTP) complex to the 5’ end of the 47S pre-rRNA belongs among the earliest steps of SSU processome formation. t-UTPs strictly co-localize with the Pol I transcription machinery; forming bead-like structures during active transcription in the nucleolus [46]. Subsequently, t-UTPs and other SSU processome factors initiate the early processing steps of 18S rRNA [46]. Importantly, a cleavage in the ITS1 region separates the processing pathways of the two subunits (for more information on the topic of rRNA processing refer to one of the following reviews [3,47]).

The maturation of rRNA is coordinated mainly by box C/D and box H/ACA small nucleolar ribonucleoprotein complexes (snoRNPs), named after a specific motif of the RNA component, which catalyze site-specific 2’-*O*-methylation and pseudouridylation of rRNA species respectively. Box C/D snoRNPs are composed of the methyltransferase fibrillarin (FBL), accessory proteins Nop56, Nop58, and 15.5K/NHPX along with the snoRNA component. The snoRNA hybridizes to the pre-rRNA to bring it into the proper conformation to be accessible for methylation by FBL. Furthermore, FBL’s function is not limited to the methylation of pre-rRNA, when it forms a complex with e.g. U3, U8 or U14 box C/D snoRNAs, it is also involved in chaperoning and directing the pre-rRNA for endo- and exonucleolytic cleavages [48]. Box H/ACA snoRNPs consist of the pseudouridine synthase dyskerin, the accessory proteins Nhp2, Nop10, Gar1 and the H/ACA snoRNA component [48]. Box H/ACA snoRNPs operate similarly to box C/D snoRNPs, besides their function in site-specific pseudouridylation and cleavage of rRNA, box H/ACA RNPs are also involved in other cellular processes such as: mRNA splicing, production of miRNAs and telomere maintenance [48,49].

In addition to snoRNPs, numerous other proteins (e.g. ATPases, GTPases, RNA helicases) are also implicated in rRNA processing. By chaperoning rRNA to facilitate proper folding, or by the removal of processing factors from the rRNA, these factors allow subsequent rRNA maturation steps and the assembly of RPs onto the rRNA to proceed [3]. An example of this is the multifunctional protein nucleolin (NCL), which is involved in multiple stages of ribosome biogenesis. NCL is recruited to the rRNA genes and interacts with both the promoter and the coding regions to facilitate transcription elongation by Pol I [50]. Furthermore, as a histone chaperone, NCL can bind to H2A-H2B dimers to promote their dissociation from the nucleosome and stimulate chromatin remodelers, like SWI/SNF and ACF, thereby increasing the rate of transcription [51]. NCL is also involved in rRNA maturation, as it binds to a specific site in the 5’-ETS region of the pre-rRNA and has a role in the cleavage of this site possibly by facilitating the action of its interaction partner, U3 snoRNA [52,53]. Moreover, NCL was demonstrated to interact with a subset of RPs and have an important function in the pre-ribosome assembly [54,55].

Nucleophosmin (NPM) is another multifunctional protein that is involved in ribosome biogenesis at multiple levels. Similarly to NCL, NPM is a histone chaperone, with the ability to stimulate rRNA transcription [56]. The requirement of NPM for rRNA processing was first described by Savkur and Olson in 1998. This study demonstrated that NPM is involved in the cleavage of pre-rRNA in the ITS2 region to promote the release of 28S rRNA [57]. These results were confirmed later on, as downregulation of NPM led to the impairment of this processing step [58]. Furthermore, NPM has been demonstrated to have a role in the nuclear export of RPL5 (uL18) and the pre-ribosomal subunits [59,60]. Additionally, NPM has been implicated in numerous other cellular processes such as: centrosome duplication, regulation of cell cycle and maintenance of genome stability [61].

In parallel with the rRNA processing the newly synthesized RPs are imported into the nucleus and assemble onto the pre-ribosomal subunits [3]. Since nascent RPs in the cytoplasm are readily degraded by the proteasome, their nuclear import has to occur immediately following their synthesis [62,63]. The nuclear import of RPs is an active, energy-dependent process facilitated by several proteins of the β-karyopherin family. Importin-β, transportin, RanBP5 and RanBP7 have been reported to promote the nuclear import of RPL23A (uL23), RPS7 (eS7) and RPL5 [64], while importin-11 was suggested to be a mediator of RPL12 (uL11) transport [65]. Furthermore, importin-7 was shown to participate in the nuclear import of several RPs, such as RPL4 (uL4), RPL6 (eL6) and RPL23A [66]. Once in the nucleus or nucleolus, RPs are believed to be actively involved in rRNA maturation presumably by stabilizing the secondary structure of the pre-rRNA. The incorporation of RPs into the pre-ribosome occurs in a highly hierarchical order, which correlates to the level of rRNA processing they are involved in, during either the early or late phases of maturation [3].

In addition to its synthesis, the maturation and assembly of 5S rRNA into the LSU is also exceptional. The precursor of the 5S rRNA is matured in the nucleus and is assembled shortly after maturation; adding two LSU RPs, RPL5 and RPL11 (uL5) to the structure. As a ternary complex, the 5S RNP is incorporated into the pre-60S subunit [67,68].

Similar to the nuclear import of RPs, the export of the pre-40S and pre-60S particles occurs through an energy-dependent process, which is also facilitated by β-karyopherins. Most importantly, exportin-1 is involved in the export of both of the pre-ribosomal subunits [69]. After their transport into the cytoplasm, pre-40S and pre-60S ribosomal subunits undergo the final maturation steps which include the dissociation of the remaining non-ribosomal proteins and the association of last RPs into their subunits [70]. Finally, the mature SSU and LSU particles can be joined together during translation initiation to fulfil their protein production function [71].

## Impaired ribosome biogenesis

**Figure 1:**
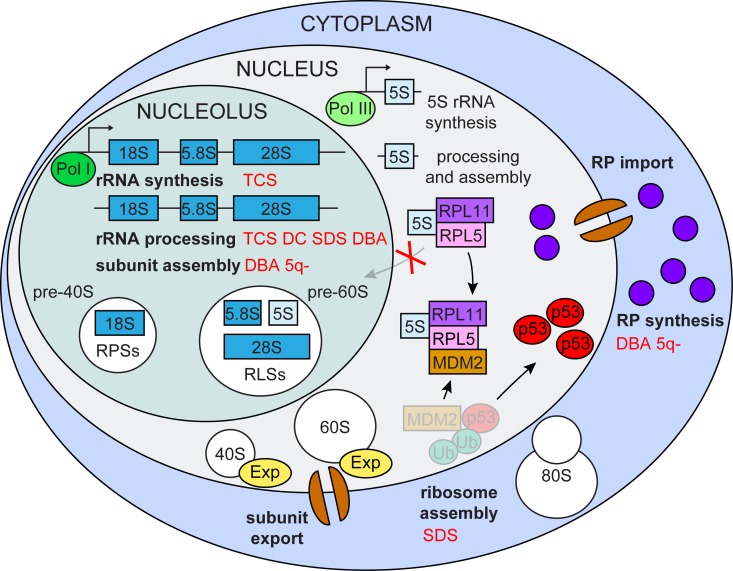
**Impaired ribosome biogenesis.** Impairment of multiple ribosome biogenesis stages (in bold black) activate p53 via the RPL5/RPL11/5S rRNA/Mdm2 pathway and is associated with various ribosomopathies (in red) TCS (Treacher Collins syndrome), DC (dyskeratosis congenital), SDS (Shwachman-Diamond syndrome), DBA (Diamond-Blackfan anemia) 5q- (5q- syndrome).

Ribosome biogenesis is an extremely energy-demanding process, which cells utilize for their growth and proliferation. In the case of impaired ribosome biogenesis, cells must immediately shut down their cell cycle to avoid incomplete growth and unprepared division. The central player in this control is the tumor suppressor protein p53 (Figure 1).

### Activation of p53 by impaired ribosome biogenesis

Under normal conditions the level of p53 in cells is kept low, despite the fact that it is continuously expressed. Downregulation of p53 is ensured post-translationally by Mdm2, an E3 ubiquitin ligase [72-74]. Mdm2 forms a heterodimer with its inactive paralogue MdmX and their interaction is required for the stability of the complex [75]. Ubiquitylation of p53 by Mdm2 stimulates the nuclear export of p53 and its degradation by the 26S proteasome [76]. In addition, the interaction between Mdm2 and p53 counteracts p53’s transactivating activity; the ability to trigger the expression of its target genes [77]. Once stabilized, p53 is also responsible for the transactivation of Mdm2, providing a negative feedback loop to quench its own activity after the activating stress has been overcome [78,79].

Perturbation of ribosome biogenesis promotes the recruitment and binding of a group of RPs and nucleolar factors to the Mdm2 central acidic domain, thereby disrupting its interaction with p53 which is then no longer degraded and thus becomes activated [8,80]. Although Mdm2 binding activity, and thus the ability to induce p53 was shown for multiple RPs, it is generally accepted that RPL5 and RPL11 have major roles in p53 activation in response to ribosomal stress. This effect is best illustrated when Pol I is inhibited by, for example, a low dose of actinomycin D (ActD) treatment, which normally induces a p53 response. In the absence of RPL5 and/or RPL11 ActD induced p53 stabilization is largely inhibited. Interestingly, depletion of other RPs cannot abolish p53 activation in this manner [81,82]. While most RPs are still synthesized during impaired ribosome biogenesis, they are rapidly degraded by the proteasome [63,82,83]. However, under these conditions RPL5 and RPL11 are able to accumulate in a ribosome free fraction, as a result of their mutual protection from proteasomal degradation, further supporting the central function of these proteins in IRBC [82]. Moreover, the assembly of RPL5 and RPL11 into the 5S RNP complex is continued even when ribosome biogenesis is impaired; the formation of this particle is essential for the binding of Mdm2 by these RPs [84]. Furthermore, the association of such a complex might render RPL5 and RPL11 more resistant to degradation when compared to other RPs.

The source of the Mdm2 binding RPs that are involved in IRBC is an intriguing question. In most cases, impairment of ribosome biogenesis leads to the disintegration of the nucleolar structure leading to spontaneous release of RPs and other nucleolar proteins into the nucleoplasm. Thus, disruption of the nucleoli seems to be an important prerequisite for p53 activation [85]. However, this logical proposal was questioned by Fumagalli et al. who demonstrated that RPS6 silencing, which inhibits SSU biogenesis, does not disrupt nucleolar structure, while p53 still accumulates via IRBC. It turned out that under these conditions translation of 5’ terminal oligopyrimidin tract containing messenger RNAs (5’-TOP mRNAs), including RPL11 and RPL5 mRNA, is upregulated [81,86]. The newly synthesized RPs are then actively imported into the nucleus to promote a p53-dependent response while nucleolar structure stays intact [8,87]. Furthermore, it has been also demonstrated that even when disintegration of the nucleoli occurs upon impaired ribosome biogenesis, the induction of p53 relies on the presence of nascent RPL5 and RPL11 proteins [82]. Thus, while disruption of the nucleolus might be only a consequence of perturbed ribosome biogenesis, the conditions and mechanisms which induce such morphological changes remain unclear.

Besides RPL5 and RPL11, there is another nucleolar factor, called alternative reading frame protein (ARF), which is capable of binding to Mdm2 and thereby promotes the activation of p53 [88]. ARF is a tumor suppressor protein encoded by the *INK4A* locus, which also encodes a cyclin-dependent kinase (CDK) inhibitor termed p16 using alternative reading frame of the same genetic locus [88,89]. Under normal conditions, ARF is expressed at low levels and sequestered into the nucleolus by NPM [90]. ARF is typically activated by oncogenes, which overstimulate ribosome biogenesis to gain excessive growth potential. Under such stimuli, ARF is released to the nucleoplasm where, similarly to RPs, it interacts with the central acidic domain of Mdm2 and indirectly promotes the stabilization of p53 [88,91,92]. Consequently, it was demonstrated that the absence of NPM triggers p53-mediated apoptosis through the activation of ARF [93]. Additionally, excessive quantities of ARF was shown to promote the degradation of NPM and therefore inhibit ribosome biogenesis [58]. This was suggested to induce 5S RNP mediated IRBC, implicating an interplay between the two pathways [87]. Moreover, ARF has a direct inhibitory effect on ribosome biogenesis; by suppressing the phosphorylation of UBF and the nucleolar import of TTF-1 it is able to shutdown rRNA synthesis, which triggers IRBC [87,94,95]. Surprisingly, one study demonstrated that overexpression of NPM also promotes the upregulation of p53, since NPM is also capable of interacting directly with Mdm2 to prevent p53’s degradation [96]. Overexpression of NPM also inhibits the translocation of p53 from the nucleus to mitochondria, which prevents the activation of the so called intrinsic apoptotic pathway [97]. However, upon apoptotic stimuli, NPM display pro-apoptotic activity as it translocates to the cytoplasm, where NPM binds the pro-apoptotic BAX protein, triggering cytochrome-c release from the mitochondria [98]. This dual function of NPM in the apoptotic process depicts the numerous functions of NPM in cells, which often differ depending on the conditions.

It is also worth mentioning that several studies have uncovered that activated IRBC also promotes cell cycle arrest through p53-independent pathways. For instance, RPL11 is capable of promoting the degradation of E2F-1 by binding to Mdm2 [99,100]; E2F-1 is a transcription factor that is required for cell cycle progression [101]. Since nearly half of human cancers have inactivated p53 [102], discovering p53-independent pathways of IRBC, makes ribosome biogenesis relevant therapeutic target in cancer research (for more detailed reviews see [11,87,103,104],).

### Impaired rRNA synthesis

Perturbation of rRNA synthesis at multiple levels was shown to activate IRBC. It has been demonstrated by numerous studies that the induction of IRBC and the stabilization of p53 can be achieved by different conditions of inhibited Pol I transcription, including: the silencing of *POLR1A*, a gene encoding the catalytic subunit of Pol I [105]; knockout of the *TIF1-A* gene [106]; or inactivation of UBF by a monoclonal antibody [85]. Impairment of the Pol I transcription machinery can also be accomplished by using several small molecule inhibitors. For instance, the DNA intercalating agent ActD is a very potent inhibitor of rRNA synthesis; it intercalates into the DNA at guanosine-cytosine (GC) rich regions which are mainly present in rDNA genes. Therefore, at lower concentrations it preferentially inhibits transcription by Pol I [107,108]. Several studies showed that ActD causes severe stress through this mechanism, disrupts the nucleolar structure and strongly induces p53 [11,85,104]. BMH-21, a newly identified drug has a similar mechanism of action, as it also intercalates into the GC-rich rDNA. Besides its incorporation into the rDNA, BMH-21 also promotes the proteasomal degradation of Pol I [109,110]. Other chemical compounds employ different mechanisms to suppress rRNA synthesis. CX-3543 (quarfloxin) inhibits transcription elongation by disrupting the interaction of NCL with rDNA [111], and CX-5461 prevents the recruitment of SL1 to rDNA promoters [112]. Both drugs are potent inducers of the IRBC response. Furthermore, CX-5461 showed a preferential toxicity in some cancer cells compared to normal primary cells, causing p53-dependent apoptosis in Eµ-*Myc* lymphoma cells [113], as well as inducing p53-independent senescence and autophagy in solid tumor cell lines [112]. CX-5461 quickly advanced to phase I clinical trials [113-115], representing an example of therapeutic potential in targeting ribosome biogenesis. Of note, a recent study showed that in addition to their inhibitory effect on rDNA transcription, both CX-5461 and CX-3543 elicit cytotoxicity through induction of DNA damage [116]. Mechanistically, these drugs bind to and stabilize the four stranded DNA structures, G-quadruplexes (G4), thereby causing replication-dependent DNA damage [111,116]. Elimination of G4 structures is carried out mainly by the homologous recombination (HR) machinery, therefore cancer cells deficient in HR components are particularly sensitive to these drugs [116]. Thus, besides the activation of IRBC, DNA damage induction also contributes to the increased sensitivity of cancer cells towards CX-5461 and CX-3543.

Impairment of the Pol III transcription machinery was also investigated by several research groups. Depletion of the *POLR3A* gene, which encodes the catalytic subunit of Pol III, impairs 5S rRNA biosynthesis and leads to cell cycle arrest in a p53-independent manner [117]. Since 5S rRNA is the essential component of 5S RNP, formed during both intact and impaired ribosome biogenesis, perturbation of its biosynthesis diminishes the formation of the ternary RNP complex which is involved in p53 stabilization. This may explain the lack of p53 induction in Pol III depleted cells [84]. Furthermore, deficiency of TFIIIA, which is involved exclusively in 5S rRNA transcription [118,119], also led to p53-independent cell cycle arrest and could reverse the activation of p53 induced by Pol I depletion, supporting the hypothesis that 5S rRNA is essential for the induction of p53 in IRBC [68,84,117,120].

Consequences of impaired rRNA synthesis and activated IRBC are well represented by patients suffering from Treacher Collins syndrome (TCS). TCS is a severe craniofacial disease with symptoms including: micrognathia, retrognathia, coloboma of the lower eyelids, loss of medial eyelashes, external ear aplasia or microtia, a large or protruding nose and zygomatic bone hypoplasia [121,122]. TCS is an autosomal dominant disorder mainly caused by mutations in the *TCOF1* gene. A minority of TCS cases (~8%) are associated with mutations in the *POLR1C* and *POLR1D* genes, which encode the RPAC1 and RPAC2 proteins, respectively. Both RPAC1 and RPAC2 proteins are parts of the RNA polymerase I and III complexes [123,124]. The *TCOF1* gene encodes a protein named Treacle, which has a prominent role in both rRNA synthesis and the early processing steps [125,126]. Haploinsufficiency of Treacle disrupts ribosome biogenesis, leading to the activation of IRBC and the initiation of p53-mediated apoptosis specific to the neural crest cells during early embryogenesis. The affected stem cell population is responsible for the formation of the bone, cartilage and connective tissue of the head [127,128]. The strong connection of IRBC and p53-induced neural crest cell apoptosis with the pathogenesis of TCS was shown in the mouse model of the disease. Similarly to TCS patients, Treacle haploinsufficient mice display severe craniofacial abnormalities. Importantly, this phenotype can be reversed either by the chemical inhibition or genetic inactivation of p53 [129]. Recent findings suggest that TCOF1 is involved in the DNA damage response (DDR) and this might also contribute to TCS pathology. It was shown by several groups that upon DNA damage DDR protein NBS1 is relocated to the nucleolus, where it interacts with TCOF1 in a CK2- and ATM-dependent manner in order to suppress rRNA transcription [130,131]. Interestingly, neuroepithelial cells, including progenitors of neural crest cells, have been reported to exhibit increased amount of DNA damage in a *Tcof1^+/-^* background. The accumulation of DNA damage has been suggested to be a consequence of the higher level of reactive oxygen species (ROS) produced in this tissue [132]. Since ROS are potent inducers of DNA damage [133,134], proficient expression of TCOF1 in neural crest cells is essential. Indeed, the administration of the antioxidant N-acetyl-cysteine partially reduced craniofacial malformations in *Tcof1^+/-^* mouse embryos and accumulation of p53 [132], indicating that both DNA damage and the IRBC contribute to TCS pathology. Additionally, a recent study provides insight into pathogenesis and tissue-specificity of TCS. Calo et al. reported that upon TCOF1 depletion the nucleolar RNA helicase DDX21 redistributes to the nucleoplasm, leading to the inhibition of ribosome biogenesis [135]. Interestingly, such disruptions in the localization of DDX21 seem to be specific for cranial neural crest cells and depletion of DDX21 alone has been presented to induce craniofacial malformations [135]. The authors suggest that rDNA damage that occurs as a consequence of impaired Pol I transcription machinery induces p53 activation and DDX21 relocalization, followed by apoptosis in tissues, which are hypersensitive to elevated levels of p53 [135]. These findings add novel layers to the research of ribosomopathies and offer new therapeutic avenues for the small group of TCS patients.

### Impaired rRNA maturation

rRNA processing is initiated co-transcriptionally and early processing factors, such as the t-UTP complex and Treacle, have been shown to have an important role in facilitating both rRNA synthesis and maturation. Therefore, perturbation of ribosome biogenesis due to the absence of these early processing factors leads to a drop in rRNA synthesis and impaired rRNA processing as well [46,125,126]. We have recently demonstrated that the depletion of one such early factor, HEAT repeat containing 1 (HEATR1) activates IRBC. Impaired expression of HEATR1 strongly induced p53 and p53-dependent cell cycle arrest. In this scenario activation of p53 was triggered by IRBC, evidenced by the robust disruption of the nucleolar structure and the emergence of Mdm2-RPL5 interaction. Furthermore, under these conditions p53 induction can be reversed by concomitant depletion of RPL5 or RPL11 [136]. UTP10, the yeast homolog of HEATR1 is a member of the t-UTP complex and has been demonstrated to have a role in rRNA synthesis as well as in early steps of pre-rRNA processing [137-139]. Correspondingly, we and others have demonstrated that human HEATR1 positively regulates rRNA synthesis and co-localizes with the Pol I transcription machinery regardless of active transcription [46,136]. Upon impaired rRNA synthesis, HEATR1, along with other Pol I associated factors, is redistributed to the periphery of the nucleolus to form so-called nucleolar caps; structures characteristic for impaired rDNA transcription [46,136,140]. Moreover, this localization appears to be solely dependent on UBF [46]. In addition, similarly to UTP10, HEATR1 has also been shown to be involved in the early 18 S rRNA maturation [46]. The exact function of HEATR1 in rRNA synthesis and processing remains largely unknown. However, as it possesses a C-terminal HEAT repeat, a motif suggested to mediate protein-protein interactions, HEATR1 might promote connections between the Pol I transcription machinery and rRNA processing factors. Analogous results, i.e. repressed transcription and processing of rRNA and IRBC activation, were obtained for other yeast t-UTP homologs, such as: 1A6/DRIM [141], WDR43 [142] and NOL11 [143].

Depletion, mutation or overexpression of numerous subsequent processing factors have been shown to impair rRNA maturation and induce IRBC [144-147]. Downregulation of the box C/D snoRNP component FBL is one such an example; it has been shown to impair rRNA processing and activate the IRBC pathway which leads to p53-mediated apoptosis in embryonic stem cells [148]. Similarly, depletion of box C/D snoRNAs, such as U3 and U8 has been proposed to induce IRBC, resulting in a very potent induction of p53 [149]. Both, FBL and U3 or U8 expression has been shown to be upregulated in several cancer types, indicating their potential involvement in tumorigenesis [149-153]. High FBL expression led to the alteration of the 2’-*O*-methylation pattern of rRNA and translational infidelity. Moreover, the altered methylation of the rRNA also promoted the internal ribosome entry site (IRES)-dependent translation of proto-oncogenic mRNAs, such as IGF1R, MYC, FGF1/2 and VEGFA [154]. An abnormal rRNA methylation pattern has been observed in aggressive breast cancer, where it induces a decrease in the IRES-dependent translation of p53, which contributes to tumor progression [153]. Additionally, opposing these effects, p53 was demonstrated to counteract such harmful methylation pattern by directly inhibiting the expression of FBL [154] Consistently, recent study by Sharma et al. showed that p53 depletion results in a robust increase in the level of FBL and introduces alterations in the methylation pattern of rRNAs. In addition, FBL ablation promotes the loss of mainly peripheral 2-O-methylated sites [155].

Mutations of the box H/ACA snoRNP component dyskerin encoding gene *DKC1* is associated with a rare genetic condition known as X-linked form of dyskeratosis congenita (X-DC). Dyskeratosis congenita (DC) is a premature aging syndrome characterized by the classical triad of mucocutaneous symptoms: abnormal pigmentation of the skin, nail dystrophy and leukoplakia of the oral mucosa. The most common cause of death is bone marrow failure, but further symptoms may also include: pulmonary fibrosis, increased risk for various malignancies, mental retardation, ophthalmic, skeletal, gastrointestinal and genitourinary abnormalities [156,157]. The pathogenesis of DC was originally thought to be a consequence of impaired rRNA processing, caused by mutations of dyskerin [49]. However, dyskerin is also a component of the telomerase complex, formed from the box H/ACA telomerase RNA component (TERC), telomerase reverse transcriptase (TERT) and the box H/ACA snoRNA associated proteins [49,156]. Patients with X-DC show accelerated telomere shortening, which mainly affects the rapidly dividing stem and progenitor cell populations. The possibility that DC is actually a telomerase dysfunction disorder is supported by the occurrence of DC due to mutations of TERT and TERC in the autosomal dominant form of the disease [49,156,158]. Furthermore, while depletion of dyskerin in human fibroblasts leads to early activation of p53, presumably through the IRBC pathway, similar upregulation of p53 was only observed later in the fibroblasts of patients with X-DC or autosomal dominant DC [159]. However, in the latter case p53 activation is actually the result of DNA damage arisen from telomere attrition after cells go through several cycles of population doubling [158,159]. In agreement with this, most of the mutations in *DKC1* gene affect the RNA binding domain, which is involved in association with TERC, rather than affecting catalytic activity or the expression level of dyskerin in X-DC cases [156,160]. *DKC1* mutations also seem to cause altered rRNA pseudouridylation, which impairs the IRES-dependent translation of a specific group of tumor suppressor mRNAs, including: p53, the CDK inhibitor p27 and the anti-apoptotic proteins XIAP and BCL-X_L_. Thus, impaired rRNA processing might contribute to the cancer susceptibility observed in X-DC patients [161,162]. In addition, similarly to FBL, the overexpression of dyskerin has also been associated with cancer [163,164], likely contributing via the dysregulated rRNA pseudouridylation, but precise mechanism is not known.

Due to their importance in ribosome biogenesis, depletion of the multifunctional proteins NCL or NPM impairs this process at multiple levels; in the case of NCL, it has been demonstrated to result in the activation of p53, presumably via IRBC [51,165]. Importantly, overexpression of NCL has been documented in many types of cancer [166]. This upregulation of NCL might promote tumorigenesis by increasing the rate of rRNA transcription and thus enhance ribosome production [167-169]. Apart from that, NCL was shown to also be involved in other cellular processes such as: chromatin organization, DNA and RNA metabolism, angiogenesis, cytokinesis, telomere maintenance, cell growth and proliferation, all of which can contribute to the tumorigenic potential of upregulated NCL [166,167,170]. Due to its high expression level NCL represents an interesting target for cancer therapy [167]. Indeed, aptameric compound AS1411, a G-rich oligonucleotide which binds to NCL with high affinity, counteracts NCL’s RNA binding activity and induces apoptosis in various cancer cells [171,172]. The therapeutic potential of AS1411 was already presented in a phase I trial for patients with different kinds of advanced cancer [173,174] and phase II trials for patients with advanced renal cell carcinoma and acute myeloid leukemia (AML) [175,176].

In contrast to other nucleolar processing factors, by binding to Mdm2, NPM has been shown to be actively involved in IRBC [96]. While another study reported that ablation of NPM also induces the upregulation of p53 through the activation of ARF [93]. Consistent with these rather conflicting results, NPM has been demonstrated to display both pro-oncogenic and tumor suppressive functions during tumorigenesis [61,177,178]. Overexpression of NPM has in fact been reported in many types of solid tumors [179-189]. Its role in tumorigenesis is commonly linked to its function in ribosome biogenesis. Interestingly, low levels of NPM have also been reported for certain cancers; such as gastric or breast cancer [190,191]. Furthermore, mutations and rearrangements of the *NPM1* gene are often seen in numerous hematological malignancies [177,192,193]. The involvement and importance of NPM in tumorigenesis, particularly in cases when it is upregulated, makes it an attractive target for cancer therapy. Indeed, several small molecule inhibitors of NPM have been tested in preclinical studies and clinical trials [194]. One such promising compound is NSC348884 which, by binding to NPM, is able to dissociate ARF from the complex with NPM; thereby inducing the upregulation of p53, which subsequently triggers apoptosis [195]. Furthermore, this compound has been shown to efficiently induce cytotoxicity in preclinical studies involving solid and hematological cancers [195,196], however clinical trials of NSC348884 has not been initiated to date.

### RP imbalance and impaired pre-ribosome assembly

The activation of p53 via the downregulation of both SSU and LSU RPs has been consistently demonstrated by multiple studies [81,82,86,197-204]. Phenotypic consequences of the RP deficiency are well represented by a rare autosomal dominant disorder called Diamond Blackfan anemia (DBA), which is a bone marrow failure syndrome due to elevated apoptosis of the erythroid progenitor cells [202,205,206]. Patients suffering from DBA often show other symptoms as well, including: short stature, craniofacial, cardiac or genitourinary abnormalities and predisposition to cancer [157,206]. Mutations in a subset of both 40S and 60S RP genes are observed in approximately 50% of DBA cases; the molecular background of the remaining cases is unknown [206-208]. In the most cases of DBA, disruption of the *RPS19 (eS19)* gene is observed, however several patients show mutations of: *RPL5*, *RPL11*, *RPL15 (eL15)*, *RPL36 (eL36)*, *RPL35A (eL33)*, *RPS7*, *RPS10 (eS10)*, *RPS17(eS17)*, *RPS24 (eS24)*, *RPS26 (eS26)* or *RPS27A (eS31)* genes. These mutations cause the haploinsufficiency of the certain RP and most likely impair the global translational capacity of the cells [205,207]. In erythroid progenitors such insufficiency reduces the production of hemoglobin, leading to increased amount of free heme which has strong pro-oxidative potential. Elevated oxidative stress then leads to p53-independent apoptosis of these cells [209,210]. This theory was well supported by a mouse model where the gene for Feline Leukemia Virus Subgroup Receptor 1 (FLVCR1), a heme exporter protein, was deleted. FLVCR1-null mice exhibit increased intracellular heme and show a phenotype resembling DBA [211]. Since the RPs which are commonly mutated in DBA patients are involved also in several diverse steps of ribosome biogenesis, their reduced expression also activates the IRBC and subsequent p53-dependent apoptosis [21,210]. Such IRBC activation is indeed detectable as accumulation of p53 has been shown in DBA-patients’ bone marrow samples [202]. Similarly, some mouse and zebrafish models of DBA, which show a similar, but not completely overlapping phenotype with impaired erythropoiesis, also have upregulated p53 [212-215]. The contribution of IRBC- and heme-induced apoptosis to the resulting DBA phenotype was studied by p53 inactivation in various models. While in zebrafish p53 inactivation only rescued developmental abnormalities, but did not affect the observed defective erythropoiesis, in mouse models inactivation of p53 reversed the apoptosis of erythroid progenitors [212,214,215].

Another ribosomopathy characterized by the reduced expression of an RP is 5q^–^ syndrome, which is often referred to as a somatically acquired form of DBA. The 5q^–^ syndrome is a myelodysplastic disease, which is predominantly present in women of advanced age and is caused by the deletion of the long arm of chromosome 5. Similarly to DBA, it is also characterized by disrupted erythropoiesis in the bone marrow, causing macrocytic anemia and a predisposition to AML. Although the extensive deletion of chromosome 5 q arm results in the loss of about 40 genes, *RPS14 (uS11)* seems to be particularly important for the pathogenesis of the disease [205,216-219]. This is illustrated by mouse models with haploinsufficiency of *RPS14* which recapitulate the human phenotype and also show upregulation of p53. In these mouse models, genetic inactivation of p53 was sufficient to rescue apoptosis of bone marrow progenitors [219]. Additionally, an increased level of p53 was also represented in hematopoietic cells of 5q^–^ patients [202,217].

Overall, due to the involvement of RPs in ribosome biogenesis a decrease in their expression leads to the initiation of the IRBC pathway. The subsequent stabilization and activation of p53 resulting in p53-dependent apoptosis seems to be the main cause of the pathogenesis of these diseases. However, active IRBC alone does not explain the tissue-specific effects of defective RPs in either of the aforementioned diseases. The sensitivity of erythroid progenitors is explained by an increased dependence on ribosome biogenesis due to rapid cell division combined with additional oxidative stress caused by the heme overload [21,209]. The relative contribution of IRBC *versus* oxidative stress to the apoptosis of erythroid progenitors remains an unanswered question.

In contrast to the decreased expression of RPs, the selective overexpression of certain RPs has been observed in multiple types of cancer, suggesting an active role in tumorigenesis [6]. For instance, RPS13 (uS15) and RPL23 (uL14) were shown to be upregulated in gastric cancer contributing to the multidrug resistance of these cells [220].

### Impaired RP import and pre-ribosome export

Golomb et al. demonstrated that depletion of β-karyopherin importin-7, not only disrupts the nuclear import of some RPs, but also causes the disruption of the nucleolar structure and activates IRBC, leading to p53 stabilization and activation [221]. In addition to β-karyopherins, other transport adaptor proteins might also be involved in the nuclear import of RPs. Lately, symportin-1 was identified as a crucial protein required for the co-import of RPL5 and RPL11 in yeast [222]. Furthermore, symportin-1 in *Chaetomium thermophilum* might also serve as a molecular chaperon for the assembly of RPL5 and RPL11 with 5S rRNA, to form the 5S RNP complex, which is able to subsequently incorporate into the LSU [223]. Whether human homolog of symportin-1, HEAT repeat containing protein 3 (HEATR3), has analogous functions remains to be investigated. Since 5S RNP is the main mediator of IRBC (as discussed above), impairment of the chaperoning of this complex might counteract the activation of the IRBC pathway and as a consequence could potentially lead to tumorigenesis.

Depletion or leptomycin-B-mediated pharmacological inhibition of exportin-1 inhibits the nuclear export of the premature subunits, induces morphological alterations of the nucleolus and activates p53 through IRBC [221]. Therefore, the disruption of either the import of RPs or the export of the pre-ribosomal particles is able to elicit the IRBC response.

As with the other steps of ribosome biogenesis, the transport of RPs and pre-ribosomal subunits also appears to be upregulated in cancer. For instance, the nuclear import of RPs was reported to be upregulated by the mTOR and c-Myc oncogenic pathways [221,224]. Moreover, c-Myc is also required for the upregulation of exportin-1 expression [221]. Thus, targeting β-karyopherins involved in ribosome biogenesis might be an appealing approach for cancer therapy; although, it is important to bear in mind that these transport adaptor proteins have a large subset of cargo proteins which are involved in other cellular processes as well.

### Impaired assembly of ribosomal subunits

One of the most important steps to initiate the subunit assembly is the release of the eukaryotic translation initiation factor 6 (eIF6) from the LSU, which is promoted by the GTPase activity of elongation factor like-1 (EFL1). Interestingly, ribosome maturation is abrogated at this step in a human autosomal recessive disorder, called Shwachman-Diamond syndrome (SDS) [225-227]. SDS is another bone marrow failure syndrome, with additional symptoms, including: exocrine pancreatic insufficiency, gastrointestinal, skeletal, immune system abnormalities and predisposition to AML [208,228,229]. Biallelic mutations in the *SBDS* gene is present in 90% of SDS cases. Ribosome maturation protein SBDS is required for the EFL1-promoted removal of eIF6 from the 60S ribosomal subunit, thus governing the final assembly of the ribosome [225-227]. Furthermore, SBDS was also reported to localize into the nucleolus [230], where it interacts with the 28S rRNA and NPM, which implies that it might have additional functions in the earlier steps of ribosome biogenesis as well [231]. The involvement of SBDS in both early and late steps of ribosome biogenesis is consistent with the observation of upregulated p53 in SDS patient-derived samples, presumably a consequence of active IRBC [232,233]. However, concomitant depletion of p53 in zebrafish and mouse models of SDS only partially rescues the pathologic phenotype; indicating that insufficient translation, alongside with activated IRBC and upregulated p53, has a prominent role in the pathogenesis of the disease [234,235].

## Aberrant ribosome biogenesis and aging

Numerous studies presented a direct connection between dysregulated ribosome biogenesis and aging. For instance, the downregulation of ribosome biogenesis components or nutrient sensing pathways, which stimulate ribosome production, have been shown to increase the lifespan of multiple organisms including *C. elegans*, *D. melanogaster,* yeast, mice and human [236-249]. Therefore, enhanced ribosome biogenesis, visualized by enlarged nucleoli, is believed to accelerate aging. Indeed, consistent with this idea, the size of the nucleoli and the amount of rRNA increases during aging in human primary fibroblasts and a single, large nucleolus is often observed in senescent cells [250,251]. Furthermore, fibroblasts isolated from patients suffering from the premature aging disease Hutchinson-Gilford progeria, have enlarged nucleoli and upregulated ribosome biogenesis [251]. Since the rate of protein translation is proportional to the rate of ribosome biogenesis [22,252] it was suggested that upregulation of protein synthesis and disruption of global proteostasis is the mechanism through which ribosome biogenesis promotes aging [253]. This theory is supported by studies showing that reduction in the rate of translation can increase lifespan, and furthermore that altered proteostasis is a hallmark of aging [238,254-258]. Additionally, caloric restriction that has been shown to promote longevity [259-261], leads to the downregulation of ribosome biogenesis by several mechanisms [262-264]. Under such dietary conditions, deacetylase SIRT1 is induced [265,266]. SIRT1, as a component of the energy dependent nucleolar silencing complex (eNoSC), is responsible for the epigenetic silencing of rDNA gene expression [264] and its overexpression can extend the lifespan [267]. Furthermore, a higher rate of metabolism and reduced amount of the tumor suppressors p53 and ARF might also contribute to aging [268,269].

### Accumulation of DNA damage in rDNA

Besides direct changes in rDNA expression level and/or rate of ribosome biogenesis, another theory relates to the accumulation of rDNA damage for aging. The repetitive nature of rDNA and the high rate of rRNA synthesis cause the rDNA repeats to be subject to recombination events and DNA damage, possibly due to collisions between the replication and transcription machineries and R-loop formations [270-274]. As a result, DNA damage can accumulate in rDNA, this in turn can lead to genome instability, which has also been implicated in cellular aging [258,275]. Indeed, it has been recently demonstrated that hematopoietic stem cells, which are highly proliferative, and thus have upregulated ribosome biogenesis, accumulate DNA damage in their rDNA genes during aging [276]. Moreover, premature aging diseases, such as Bloom and Werner syndromes are associated with increased rDNA instability [277-279]. BLM and WRN helicases, that are mutated in Bloom and Werner syndromes, respectively have been shown to associate with the Pol I transcription machinery and promote rRNA synthesis [280,281]. These findings indicate that rDNA instability in these diseases can be attributed to disrupted rRNA transcription and consequent accumulation of rDNA damage due to unresolved rDNA structures.

### Deregulation of ribosome biogenesis in aging

Several studies have reported the downregulation of ribosome biogenesis in aged tissues. A progressive decrease in the expression of RPs or rRNA has been observed during the aging process [282,283], inefficient ribosome biogenesis has been accounted for age-related cataract [284] and diminished skeletal muscle hypertrophy [285]. On the other hand, it has been suggested that such decrease of ribosome biosynthesis may be a compensatory mechanism in aged tissues to prolong lifespan [283].

Being an age-related disease, upregulation of ribosome biogenesis and increased size of the nucleoli have been observed in various types of cancer cells [18]. Numerous reports suggests that rather than being a passive consequence of tumorigenesis, upregulation of ribosome biogenesis is a key step to promote this process [113,162,286]. The increase in the rate of ribosome biogenesis drives translation, excess growth and proliferation [287] and the selective upregulation of certain ribosome biogenesis components in many cases contributes to tumorigenesis. For instance, overexpression of key rRNA processing factors, such as FBL or dyskerin has been reported in various cancers [150-153,163,164]. Upregulation of FBL or dyskerin alters the posttranscriptional modification of the rRNAs, thus changes the structure of the ribosomes. These altered ribosomes presumably do not change the amount of total protein production, however they affect the quality of translation [288]. Marcel et al. designated these altered complexes ‘cancer ribosomes’ in FBL upregulated cells, because of their active involvement in tumorigenesis due to preference for IRES-dependent translation of oncogene mRNAs [154]. Moreover, FBL overexpression has been observed in aged mice [289] and lower expression of it seems to be associated with increased lifespan in humans [262]. Additionally, similarly to FBL and dyskerin, selective overexpression of certain RPs has been reported to promote tumorigenesis [220,290,291]. Changes in the balance of the RPs might change the structure of the ribosome; however, since many of these RPs possess extra-ribosomal functions, these cannot be excluded from contribution to tumorigenesis.

A high rate of ribosome biogenesis and enlarged nucleoli are the main characteristics of stem cells as well as cancer cells. Similarly to cancer cells, stem cells rely on ribosome biogenesis for their growth and proliferation and it also ensures pluripotency [148,292-294]. During differentiation these cells lose high expression of ribosome biogenesis factors and obtain shrunken nucleoli [295]. Several studies have demonstrated that partial depletion of certain nucleolar factors involved in ribosome biosynthesis induces differentiation of pluripotent stem cells [148,292,294,296,297]. Furthermore, complete loss of some ribosome biogenesis components affects stem cells more drastically, when compared to differentiated cells [148,297]. Consistently, decreased expression of ribosome biosynthesis factors observed in ribosomopathies induces growth arrest and apoptosis in hematopoietic or other stem cell types, while differentiated cells remain mostly unaffected. Furthermore, although upregulation of ribosome biogenesis is traditionally associated with aging and cancer, downregulation of this process can also promote tumorigenesis, as patients with ribosomopathies are predisposed to development of certain cancer types [20,205,208]. This can be explained as a result of a lower amount of available mature ribosomes introducing competition between various mRNAs. Thus tumor suppressors encoding mRNAs with lower affinity to the ribosome may lose their translational capacity [287]. High and stable expression of p53 can decrease lifespan in mice and humans [298-300], therefore it is possible that upregulated p53 usually observed in ribosomopathies can also contribute to accelerated aging of those patients. Indeed, one of the ribosomopathies, dyskeratosis congenita has been associated with premature aging. Whether this is a more general feature that is also shared by other ribosomopathies needs further investigation. Although, both upregulation and downregulation of ribosome biogenesis can accelerate aging process, timing of the downregulation of the ribosome biogenesis is important factor that must be considered. While numerous studies show that an overall decrease in ribosome biogenesis promotes longevity, it must occur in the post-developmental phase. When it is downregulated early in life, as in the case of ribosomopathies, it has more severe consequences, which reduce lifespan [301].

Although differentiated, non-dividing cells usually display shrunken nucleoli and reduced rate of ribosome biogenesis, prominent nucleoli can be observed in terminally differentiated neurons [17]. It has been demonstrated that during development, post-mitotic neurons rely on increased ribosome biogenesis for their somatoneuritic growth [302,303]. Specifically, neurotrophics, such as the brain-derived neurotrophic factor (BDNF) stimulate ribosome biosynthesis, through the ERK1/2 signaling cascade [302]. Consequently, upregulated ribosome biogenesis supply developing neurites with a sufficient number of ribosomes for the increased local protein synthesis to promote morphogenesis of the neurons [17,302]. Furthermore, it has been also suggested that neurite outgrowth, which is promoted in mature neurons during regeneration of the nerves following injury, depends on the upregulation of ribosome biogenesis [304,305].

### Ribosome biogenesis and neurodegenerative diseases

The importance of active ribosome biogenesis in mature neurons is further supported by the observation that it is frequently impaired in neurodegenerative diseases. For instance, Alzheimer’s disease (AD) has been reported to associate with reduced number of the ribosomes [306], which may be the linked to the increased oxidation of rRNA [307,308] and/or epigenetic silencing of rDNA, seen in AD patient’s brains [309,310]. Furthermore, aberrant NORs have been also observed in AD patients [311]. Additionally, the microtubule-associated protein, tau, whose function is severely impaired in AD, has been reported to localize to the nucleolus, where it interacts with several nucleolar proteins and may have a role in several nucleolus-associated functions under normal conditions [312-315]. Downregulation of ribosome biogenesis has also been documented in Parkinson’s disease (PD), which is often accompanied with disrupted nucleolar structure of the affected dopaminergic neurons [316,317]. This phenotype may be mediated by NCL, since its expression has been reported to be decreased in the *substantia nigra* of PD patients [318]. Furthermore, NCL has been also documented to interact with α-synuclein and DJ-1, the two major proteins involved in the pathogenesis of familial PD [319]. Moreover, a mutation of DJ-1 has been presented to impair ribosome biogenesis by the exclusion of TNF receptor associated protein (TTRAP) from the nucleolus [320]. Whereas another study on PD has been reported that the overexpression of parkin associated substrate (PARIS) represses rRNA transcription by direct interaction with the Pol I transcription machinery [321]. Several factors perturbing ribosome biogenesis have been observed in Huntington’s disease (HD) as well. For instance, the PIC component, UBF has been shown to be downregulated in HD patients [322]. UBF’s function and thus rRNA synthesis has been also suggested to be inhibited via the decreased acetylation and/or increased methylation of UBF, both mediated by the mutant huntingtin protein [322,323]. Furthermore, it has been also suggested that the CAG triplet expansion containing transcripts, characteristic of HD, are able to associate with NCL and this interaction leads to the reduced recruitment and binding of NCL to the rDNA promoter, followed by promoter hypermethylation and results in the rRNA synthesis suppression [324]. Overall, numerous studies indicate that impaired ribosome biogenesis is a key feature of neurodegeneration. The diversity and complexity of mechanisms that perturb this process indicate the existence of more factors capable of impairing ribosome biogenesis in these syndromes with a rather heterogeneous genetic background. Additionally, since the accumulation of p53 has been reported in AD, PD and HD [325-327], the activation of IRBC is evident and may be fundamental for the pathology of these diseases.

Although the complex relationship between aging, age-related diseases and ribosome biogenesis and the regulation thereof is just being elucidated, the importance of the tight regulation of these processes is evident from these examples.

## CONCLUSION

In the past decades, a tremendous effort was made to explore the various steps of ribosome biogenesis and the regulation of this process. It has long been acknowledged that due to its complexity, ribosome biogenesis requires a huge energy investment from cells. Therefore, it is regulated by numerous complex pathways. The impairment of ribosome biogenesis, at any step from rRNA synthesis to ribosome assembly, has been demonstrated to result in severe consequences such as: cell cycle arrest, senescence or apoptosis mainly through the RPL5/RPL11/5S rRNA/Mdm2/p53 axis. Although the process of IRBC is well-established and widely accepted, further research is ongoing. For instance, it is not fully understood how the defects in various steps of ribosome biogenesis are sensed and transduced to uniformly induce IRBC.

The dependence of ribosome biogenesis on the nutrient and energy status of cells renders the entire process highly vulnerable to internal and external stress stimuli. Indeed, multiple studies have reported that a number of typical cellular stressors, such as: DNA damaging agents (UV- and γ-irradiation, genotoxic chemotherapeutics); hypoxia, nutrient and growth factor deprivation; heat shock and oncogene activation induce alterations in ribosome biogenesis and ultimately activate the IRBC [328]. Consistently, a report from Burger and colleagues showed that a diverse group of commonly used chemotherapeutic drugs (e.g. alkylating agents, antimetabolites, mitosis inhibitors, kinase inhibitors, translation inhibitors, etc.), are all capable of perturbing ribosome biogenesis [108]. Interestingly, the stage of ribosome biogenesis inhibition differed between these compounds; some of them suppressed the process earlier while others inhibited later steps [108]. These results suggest that chemotherapeutic agents induce IRBC, which might contribute to their cytotoxicity. IRBC-induced apoptosis or senescence might be beneficial for cancer therapeutics, since cancer cells highly rely on ribosome production for their growth and proliferation. However, traditional chemotherapeutic drugs possess other cytotoxic effects such as: genotoxicity, nucleotide deprivation, inhibition of signal transduction, and others which poison non-cancerous cells as well. Therefore, it might be more favorable to take advantage of those compounds, which are rather specific and exclusively inhibit ribosome biogenesis. However, these agents must still be treated with caution, as other populations of rapidly dividing cells, such as stem cells might be sensitive to the perturbation of ribosome biogenesis. Other therapeutic approaches, targeting the various steps of ribosome biogenesis may be a valid therapeutic strategy, as selective upregulation of some ribosome biosynthesis factors is observed in various cancers.
